# Anthropometric Clusters of Competitive Cyclists and Their Sprint and Endurance Performance

**DOI:** 10.3389/fphys.2019.01276

**Published:** 2019-10-09

**Authors:** Stephan van der Zwaard, Cornelis J. de Ruiter, Richard T. Jaspers, Jos J. de Koning

**Affiliations:** ^1^Department of Human Movement Sciences, Faculty of Behavioural and Movement Sciences, Amsterdam Movement Sciences, Vrije Universiteit Amsterdam, Amsterdam, Netherlands; ^2^Leiden Institute of Advanced Computer Science, Universiteit Leiden, Leiden, Netherlands; ^3^Laboratory for Myology, Department of Human Movement Sciences, Faculty of Behavioural and Movement Sciences, Amsterdam Movement Sciences, Vrije Universiteit Amsterdam, Amsterdam, Netherlands; ^4^Department of Exercise and Sport Science, University of Wisconsin-La Crosse, La Crosse, WI, United States

**Keywords:** physical performance, cycling, anthropometry, sports specialization, data science, machine learning

## Abstract

Do athletes specialize toward sports disciplines that are well aligned with their anthropometry? Novel machine-learning algorithms now enable scientists to cluster athletes based on their individual anthropometry while integrating multiple anthropometric dimensions, which may provide new perspectives on anthropometry-dependent sports specialization. We aimed to identify clusters of competitive cyclists based on their individual anthropometry using multiple anthropometric measures, and to evaluate whether athletes with a similar anthropometry also competed in the same cycling discipline. Additionally, we assessed differences in sprint and endurance performance between the anthropometric clusters. Twenty-four nationally and internationally competitive male cyclists were included from sprint, pursuit, and road disciplines. Anthropometry was measured and *k*-means clustering was performed to divide cyclists into three anthropometric subgroups. Sprint performance (Wingate 1-s peak power, squat-jump mean power) and endurance performance (mean power during a 15 km time trial, $\dot{\text{V}}$O_2__peak_) were obtained. *K*-means clustering assigned sprinters to a mesomorphic cluster (endo-, meso-, and ectomorphy were 2.8, 5.0, and 2.4; *n* = 6). Pursuit and road cyclists were distributed over a short meso-ectomorphic cluster (1.6, 3.8, and 3.9; *n* = 9) and tall meso-ectomorphic cluster (1.5, 3.6, and 4.0; *n* = 9), the former consisting of significantly lighter, shorter, and smaller cyclists (*p* < 0.05). The mesomorphic cluster demonstrated higher sprint performance (*p* < 0.05), whereas the meso-ectomorphic clusters established higher endurance performance (*p* < 0.001). Overall, endurance performance was associated with lean ectomorph cyclists with small girths and small frontal area (*p* < 0.05), and sprint performance related to cyclists with larger skinfolds, larger girths, and low frontal area per body mass (*p* < 0.05). Clustering optimization revealed a mesomorphic cluster of sprinters with high sprint performance and short and tall meso-ectomorphic clusters of pursuit and road cyclists with high endurance performance. Anthropometry-dependent specialization was partially confirmed, as the clustering algorithm distinguished short and tall endurance-type cyclists (matching the anthropometry of all-terrain and flat-terrain road cyclists) rather than pursuit and road cyclists. Machine-learning algorithms therefore provide new insights in how athletes match their sports discipline with their individual anthropometry.

## Introduction

The athlete’s physique is important for success in many sports (Norton et al., 1996). Even though there are many determinants that contribute to the performance of athletes, most sports require a specific range in body size and shape to compete at the top level (Norton and Olds, 2001). Consequently, athletes tend to specialize toward sports disciplines that are well aligned with their anthropometry (Foley et al., 1989). Physical comparisons of athletic champions support this anthropometry-dependent specialization, revealing large anthropometric differences between sports disciplines and a much more similar physique within sports disciplines, especially at higher levels of competition (Carter, 1970). It should be noted, however, that anthropometric measures are commonly reported for groups of a specific sports discipline (Carter, 1970; Norton and Olds, 2001), focusing on group averages and standard deviations (Norton and Olds, 2001) or distributions of a single anthropometric measure within these groups (Carter, 1970). What remains to be elucidated is whether grouping of athletes based on similarities in their individual anthropometry using multiple anthropometric dimensions, and subsequently evaluating their sports discipline, will provide new insights in anthropometry-dependent specialization.

In cycling, for example, athletes specialize into the disciplines sprint, pursuit, uphill, time trial, flat-terrain, and all-terrain, each demonstrating distinct anthropometric characteristics (Foley et al., 1989; Padilla et al., 1999; Lucía et al., 2000; Mujika and Padilla, 2001; Menaspà et al., 2012). For instance, road climbers pursue a low body mass to enhance their uphill performance, as body mass increases the resistance from gravity (Mujika and Padilla, 2001). Flat-terrain cyclists reduce their frontal area per body mass to improve performance during flat stages, minimizing relative energy costs to aerodynamic resistance (Mujika and Padilla, 2001). The diversity in body shapes is represented by the somatotypes, describing a predisposition toward specific forms of physical activities (Gabriel and Zierath, 2017). Mesomorph body shapes are beneficial for strength and speed activities, endomorphy contributes to strength and maximal force, whereas ectomorphy is advantageous for endurance and uphill performance (Gabriel and Zierath, 2017). Accordingly, sprint-type cyclists were found to have high mesomorphy, whereas endurance-type cyclists demonstrated higher ectomorphy and lower mesomorphy (Foley et al., 1989). Also in cycling, these anthropometric measures are commonly reported in averages and standard deviations for predefined groups of a specific sports specialization (e.g., Foley et al., 1989; Padilla et al., 1999; Lucía et al., 2000; Mujika and Padilla, 2001; Menaspà et al., 2012). However, these predefined groups may still include individual athletes with a dissimilar anthropometry, which would affect the group’s average anthropometry and confound the assessment of anthropometry-dependent sports specialization.

Alternatively, one could identify subgroups of athletes solely based on their individual anthropometry, and independent of their predefined sports discipline. Uncovered groups of athletes with similar anthropometry and subsequent evaluation of their actual sports disciplines will reveal whether there is an unbiased interdependence between anthropometry and sports discipline. Over the last decade, artificial intelligence has been introduced into sports science, providing new opportunities for data analytics in sports. As part of artificial intelligence, machine-learning techniques now enable us to identify subgroups of athletes with similar anthropometry, using an integrative approach with multiple anthropometric dimensions. Unsupervised machine-learning techniques, like *k*-means clustering, help researchers to discover “hidden” patterns in their data and to use these patterns to classify athletes such that athletes within one subgroup are anthropometrically similar to each other, but different from athletes in another subgroup. With the implementation of such data science techniques, it is now possible to provide a new and unbiased perspective on anthropometry-dependent sports specialization. To our knowledge, it is currently unknown whether the athletes in an anthropometric cluster that is identified by similarities in individual anthropometry using multiple anthropometric measures will also compete in the same sports discipline, which would confirm anthropometry-dependent sports specialization.

In addition to the athlete’s sports specialization, the athlete’s physical performance will help to provide a more detailed comprehension of anthropometry-dependent sports specialization. Differences in sprint and endurance performance are of interest, as it has been highlighted that performance and physiological parameters should be interpreted in the context of the athlete’s individual anthropometry (Mujika and Padilla, 2001). The relationships between anthropometric measures and athletic performance have been assessed in various sports (Chaouachi et al., 2009; Knechtle et al., 2011; Brocherie et al., 2014). Endurance performance was negatively related to sum of skinfolds in male triathletes (Knechtle et al., 2011); however, others found no relationship between anthropometric measures and track cycling performance within subgroups of cyclists (McLean and Parker, 1989). What remains to be elucidated is how anthropometry relates to both sprint and endurance performance in one heterogeneous group of competitive sprint, pursuit, and road cyclists. Anthropometric clustering using unsupervised machine learning is expected to provide a new perspective on the interrelationships between anthropometry, sports specialization, and athletic performance.

The aim of this study was to identify clusters based on individual anthropometry of sprint, pursuit, and road cyclists using multiple anthropometric measures, and to evaluate whether athletes with a similar anthropometry also competed in the same cycling discipline. Additionally, we aimed to assess differences in the anthropometric clusters’ sprint and endurance performance. Moreover, relationships between anthropometric characteristics and both sprint and endurance performance were assessed in all cyclists. We hypothesized that clustering based on anthropometry will reveal separate subgroups for sprint, pursuit, and road cyclists, confirming anthropometry-dependent specialization in cycling.

## Materials and Methods

### Subjects

Twenty-four male cyclists from sprint, pursuit, and road disciplines volunteered to participate in this study. Cyclists competed at the national, international, or Olympic level. Prior to participation, subjects were familiarized with the experimental procedures and subjects provided written informed consent. The study was approved by the medical ethics committee of the VU medical center, Amsterdam, Netherlands (NL49060.029.14) and conducted according to the principles of the Declaration of Helsinki.

### Design

In this observational study, subjects visited the lab three times. During the first visit, anthropometry was measured and subjects performed a maximal incremental exercise test. The second visit consisted of a vertical squat-jump test and 15-km cycling time trial. In the third visit, subjects performed a 30-s Wingate test. Before each visit, subjects were instructed to avoid strenuous exercise and alcohol consumption within the last 24 h and to consume no caffeine or food during the last 3 h before each test. Cycle ergometer handlebar and saddle height were adjusted individually and subjects used their own clipless pedals.

### Methodology

#### Anthropometry

Measurements of body mass, stature, skinfolds, girths, and breadths were obtained by the same investigator in accordance with the International Society for the Advancement of Kinanthropometry (ISAK) level 1 protocol (Marfell-Jones et al., 2006). All measurements were taken on the right side of the subject’s body. Skinfolds were obtained with a Harpenden skinfold caliper (Baty International, West Sussex, United Kingdom). Breadths were measured with a Cescorf sliding bone caliper, after applying appropriate pressure to minimize the influence of soft tissue. Measures were obtained in duplicate and mean values were used, or in triplicate using median values [i.e., if the first and second measure differed >5% for skinfolds or >1% for other anthropometric measures (Marfell-Jones et al., 2006)]. Somatotypes were determined according to the Heath–Carter model (Heath and Carter, 1967). Body surface area was determined from weight and height according to Du Bois and du Bois (1916), body fat percentage was derived from the sum of four skinfolds (Durnin and Womersley, 1974), and percentage skeletal muscle mass was estimated using an anthropometric regression model (Lee et al., 2000).

#### Sprint Performance

Sprint performance was assessed by the 1-s peak power output (PO_peak_) during a 30-s Wingate test on a bicycle ergometer (Monark 894 E Peak Bike, Monark Exercise AB, Vansbro, Sweden), as described elsewhere (Van der Zwaard et al., 2018). The test was preceded by a 10-min warm-up (brake weight 1.5 kg) with three short accelerations. Workload was set at 10% body mass and was automatically applied to the flywheel after two revolutions. Subjects were instructed to remain seated and received strong verbal encouragement throughout the test.

Cyclists also performed a vertical squat-jump test. Subjects were instructed to bend their knees to a 90° knee angle and hold this position for 3 s before push-off. Jumps were performed without arm swing, with hands placed above the hips. Cyclists performed four jumps, with 2-min rest in-between consecutive jumps. A fifth jump was performed if the fourth jump was >5% higher than the previous jumps. An inertial measurement unit (MPU-9150, ±16.0 g, 500 Hz, Invensense, San Jose, CA, United States) was firmly secured to the lower back, and was used to calculate average jump power during push-off. Vertically directed acceleration was multiplied by body mass to derive vertical force, which was multiplied by vertical velocity (i.e., integrated acceleration) to obtain the vertical power production. Subsequently, power production was averaged over the entire push-off phase, from the initial increase in vertical acceleration until takeoff. To ensure that analyzed jumps were actual squat jumps, the jumps with a countermovement were excluded. The highest squat jump was used for analysis.

#### Endurance Performance

Endurance performance was obtained from a 15-km time trial on an electronically braked bicycle ergometer (VU-MTO, Amsterdam, Netherlands), as described previously (Van der Zwaard et al., 2018). Gear ratio could be altered during the time trial. Mean power output was determined from torque and cadence measurements, sampled at 100 Hz and averaged over the duration of the time trial (PO_TT_).

Subjects also performed a maximal incremental exercise test to obtain peak oxygen uptake ($\dot{\text{V}}$O_2__peak_), as described elsewhere (Van der Zwaard et al., 2016). $\dot{\text{V}}$O_2_ was recorded breath-by-breath using open circuit spirometry (Cosmed Quark CPET, Cosmed S.R.L., Rome, Italy). Before every test, volume transducer and gas analyzer were calibrated according to manufacturer’s instructions. $\dot{\text{V}}$O_2_ data were filtered for extreme values and $\dot{\text{V}}$O_2__peak_ was defined as the highest average 30-s $\dot{\text{V}}$O_2_ value.

#### Unsupervised Machine Learning

*K*-means clustering is a popular unsupervised machine-learning algorithm that divides a data set into subgroups based on patterns in the data. Here, we performed *k*-means clustering with the Hartigan–Wong algorithm (Hartigan and Wong, 1979) and divided cyclists into subgroups based on anthropometric measures of body shape (meso-, ecto-, and endomorphy), body size (height, weight, and body surface area), and body composition (sum of eight skinfolds, body fat percentage, and skeletal muscle mass percentage). Optimization was performed for maximal compactness of clusters by minimizing the total within-cluster variation over all *k* clusters (Eq. 1). Initially, the algorithm provides a random cluster center for all *k* clusters. Then, observations are assigned to the nearest cluster center based on the shortest Euclidean distance, and after all data points have been assigned, the cluster centers are recalculated. The “cluster assignment” and “cluster center update” steps are iterated until the cluster assignment stops changing or the maximum number of iterations is reached.

$$\mathrm{tot}.\mathrm{withinss}=\sum_{k=1}^{K}\sum_{x_{i}\in C_{k}}{||x_{i}-\mu_{k}||}^{2}$$

Total within-cluster variation is minimized by minimizing the sum of squared error in Euclidean distance between individual data points and cluster centers. Where *x*_*i*_ is the individual data point belonging to cluster *C*_*k*_, μ*_*k*_* is the center of cluster *C*_*k*_, || *x*_*i*_ − μ*_*k*_*|| is the Euclidean distance between the individual data point and cluster center, and *K* is the total number of clusters, which must be specified before clustering.

*K*-means clustering was performed using the stats package in R. Before clustering, anthropometric measures were standardized into *Z*-scores, removing differences in measurement scales between variables. Using this input data, the appropriate number of clusters was determined by the Elbow Criterion, Bayesian Information Criterion from the mclust package (Scrucca et al., 2016), and cluster validity criterions from the NbClust package (Charrad et al., 2014), and was found to be three clusters. Maximum number of iterations was set at 50 (though clusters were obtained within three iterations). Moreover, optimization was performed using 25 random starting partitions as initial cluster centers to enhance cluster stability.

### Statistical Analysis

All data are presented as individual values or as mean ± SD. All performance measures were expressed relative to the body mass of cyclists. One-way ANOVA tests or non-parametric Kruskal–Wallis tests were used to detect group-differences between anthropometric clusters, and least significant difference *post hoc* tests or Mann–Whitney tests were used to localize differences. Pearson or Spearman correlations were used to assess relationships between anthropometry and physical performance. Differences were considered statistically significant if *p* < 0.05. Tendencies were reported if *p* < 0.10.

## Results

### Anthropometric Clusters

**FIGURE 1:**
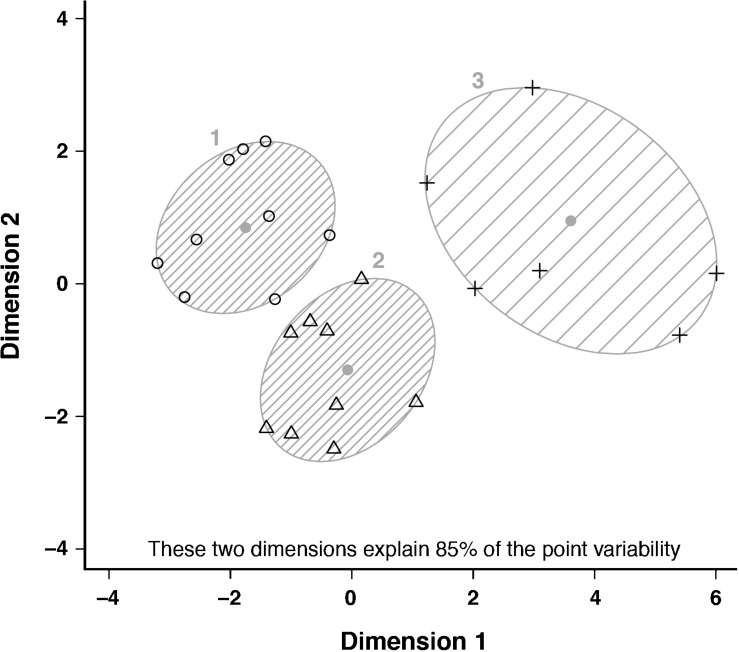
Cluster plot with a two-dimensional representation of the three anthropometric clusters. Clusters are displayed in the two most important dimensions, which represent a combination of the anthropometric characteristics and were obtained after dimension reduction of our higher-dimensional data set [i.e., dimensions explaining 85% of the variation in our data set; for more details, see Pison et al. (1999)]. Individual values, cluster centers, and spanning ellipses of clusters are presented for the short meso-ectomorph cluster (1, circles), the tall meso-ectomorph cluster (2, triangles), and mesomorph cluster (3, pluses).

**FIGURE 2:**
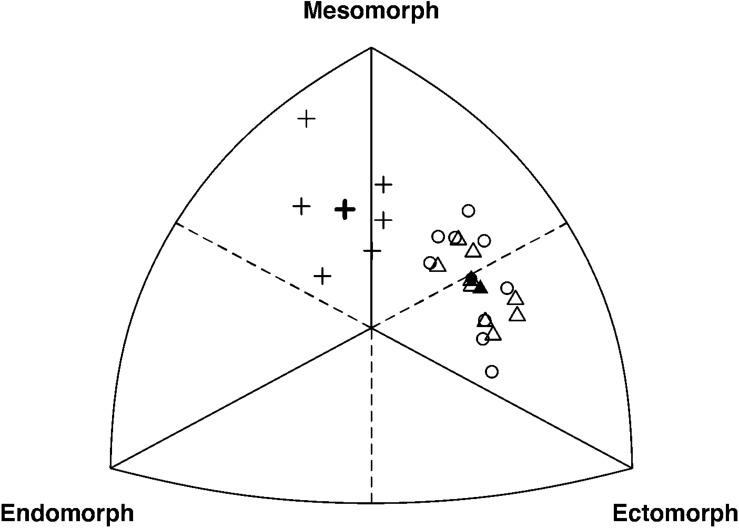
Somatochart with mesomorphy, endomorphy, and ectomorphy values of the three anthropometric clusters. Individual and average somatotype values (i.e., open and closed symbols, respectively) are presented per cluster, including the mesomorph cluster (pluses), short meso-ectomorph cluster (circles), and tall meso-ectomorph cluster (triangles).

*K*-means clustering divided cyclists into three anthropometric clusters based on individual differences in body shape, size, and composition (Figure 1 and Table 1). All sprint cyclists were allocated to a mesomorphic cluster (endo-, meso-, and ectomorphy were 2.8, 5.0, and 2.4, respectively; *n* = 6). Pursuit and road cyclists were distributed over a short meso-ectomorphic cluster (1.6, 3.8, and 3.9; *n* = 9), and tall meso-ectomorphic cluster (1.5, 3.6, and 4.0; *n* = 9). The somatochart of these subgroups is displayed in Figure 2. The mesomorphic cluster consisted of heavier cyclists with larger girths, but who were not as lean as cyclists of other clusters. These sprinters also had a lower frontal area per body mass. The short meso-ectomorphic cluster included cyclists that were significantly lighter, shorter, and smaller compared to cyclists in the tall meso-ectomorphic cluster, demonstrating lower thigh and shank lengths, smaller femur breadths, and smaller girths, but a higher percentage skeletal muscle mass. Pursuit and road cyclists were not allocated to different clusters, but were evenly distributed over the short and tall meso-ectomorphic clusters.

**TABLE 1:** Anthropometric characteristics of cyclists within the three anthropometric clusters.

**Cluster**	**Mean ± SD**			***p*-value**		
		
	**Mesomorphic**	**Tall**	**Short**	**Tall vs.**	**Short vs.**	**Short vs.**
		**meso-ectomorphic**	**meso-ectomorphic**	**meso cluster**	**meso cluster**	**tall cluster**
						
	**(*n* = 6)**	**(*n* = 9)**	**(*n* = 9)**			
**Basics**						
Age (years)	26 ± 8	26 ± 8	24 ± 8	0.859	0.634	0.375
Height (m)	1.86 ± 0.06	1.91 ± 0.03^∗^	1.81 ± 0.03^∗^^#^	0.038	0.033	<0.001
Body mass (kg)	87.6 ± 6.2	79.1 ± 3.6^∗^	68.4 ± 3.5^∗^^#^	0.001	<0.001	<0.001
BMI (kg/m^2^)	25.3 ± 1.1	21.7 ± 0.6^∗^	20.9 ± 0.9^∗^^#^	<0.001	<0.001	0.063
Cycling discipline	6 Sprint	4 Pursuit | 5 Road	4 Pursuit | 5 Road			
**Somatotypes**						
Mesomorphy	5.0 ± 0.8	3.6 ± 0.7^∗^	3.8 ± 0.9^∗^	0.003	0.009	0.631
Ectomorphy	2.1 ± 0.6	4.0 ± 0.3^∗^	3.9 ± 0.5^∗^	<0.001	<0.001	0.546
Endomorphy	2.8 ± 0.7	1.5 ± 0.4^∗^	1.6 ± 0.3^∗^	0.002	<0.001	0.489
**Body composition**						
Lean body mass (kg)	74.2 ± 3.1	71.8 ± 3.7	61.8 ± 3.7^∗^^#^	0.236	<0.001	<0.001
Lean body mass (%)	84.8 ± 3.7	90.8 ± 2.1^∗^	90.4 ± 1.9^∗^	0.005	0.003	0.605
Muscle mass (kg)	34.8 ± 1.9	33.4 ± 1.4^∗^	30.4 ± 1.4^∗^^#^	0.088	<0.001	<0.001
Muscle mass (%)	39.8 ± 1.3	42.2 ± 1^∗^	44.4 ± 1.5^∗^^#^	0.002	<0.001	0.001
Body fat (kg)	13.4 ± 4.1	7.3 ± 1.7^∗^	6.6 ± 1.3^∗^	0.002	<0.001	0.605
Body fat (%)	15.2 ± 3.7	9.2 ± 2.1^∗^	9.6 ± 1.9^∗^	0.005	0.003	0.605
**Body size**						
Body surface area	2.12 ± 0.11	2.07 ± 0.06	1.87 ± 0.06^∗^^#^	0.208	<0.001	<0.001
Frontal area	0.39 ± 0.02	0.38 ± 0.01	0.35 ± 0.01^∗^^#^	0.208	<0.001	<0.001
BSA/body mass ^∗^ 10^–3^	24.3 ± 0.6	26.2 ± 0.4^∗^	27.4 ± 0.7^∗^^#^	<0.001	<0.001	<0.001
FA/body mass ^∗^ 10^–3^	4.5 ± 0.1	4.8 ± 0.1^∗^	5.1 ± 0.1^∗^^#^	<0.001	<0.001	<0.001
**Lengths (cm)**						
Thigh	47.6 ± 2.3	50.4 ± 2.2^∗^	46.6 ± 1.8^#^	0.016	0.372	0.001
Shank	42.8 ± 2.3	43.8 ± 1.4	40.6 ± 1.1^∗^^#^	0.242	0.014	<0.001
**Skinfolds (mm)**						
Sum of six skinfolds	69.3 ± 17.7	40.1 ± 3.7^∗^	41.4 ± 6.8^∗^	<0.001	0.001	0.508
Sum of eight skinfolds	92.1 ± 22.9	52.5 ± 4.3^∗^	52.8 ± 8.4^∗^	0.002	0.002	0.536
**Breadths (mm)**						
Humerus	7.4 ± 0.1	7.2 ± 0.4	7.0 ± 0.2^∗^	0.181	0.010	0.116
Femur	10.1 ± 0.4	10.2 ± 0.4	9.8 ± 0.5^#^	0.763	0.123	0.044
**Girths (cm)**						
Upper arm relaxed	32.7 ± 2.0	29.1 ± 0.6^∗^	27.0 ± 1.3^∗^^#^	0.001	<0.001	<0.001
Upper arm flexed	34.0 ± 1.9	31.3 ± 0.9^∗^	29.1 ± 1.2^∗^^#^	0.001	<0.001	0.002
Waist	83.7 ± 4.1	77.5 ± 3.4^∗^	74.3 ± 2.2^∗^^#^	0.001	<0.001	0.043
Gluteal	103.5 ± 2.4	97.9 ± 2.0^∗^	93.2 ± 3.2^∗^^#^	0.001	<0.001	0.001
Calf	39.4 ± 2.2	37.9 ± 2.0	36.4 ± 2.1^∗^	0.184	0.012	0.139
Thigh	60.1 ± 2.5	55.9 ± 2.2^∗^	52.6 ± 1.3^∗^^#^	0.001	<0.001	0.002

*Values are mean ± SD. ^∗^ significantly different from mesomorphic cluster, *p* < 0.05. ^#^ significantly different from tall meso-ectomorphic cluster, *p* < 0.05. ^∗^ and ^#^ indicate tendencies, *p* < 0.10. BMI, body mass index; BSA, body surface area; FA, frontal area.*

### Sprint and Endurance Performance of Clusters

**FIGURE 3:**
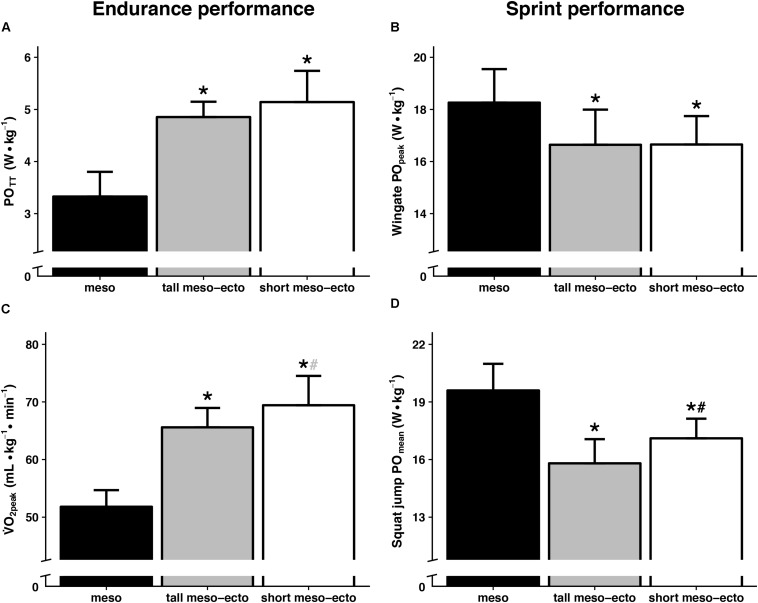
Group-differences in endurance performance (left) and sprint performance (right) were presented for the three anthropometric clusters. Time trial performance **(A)** and $\dot{\text{V}}$O_2__peak_ **(C)** were considered as measures of endurance performance, Wingate peak power **(B)** and squat jump mean power **(D)** were taken as measures of sprint performance. Data are presented as mean ± SD. ^∗^ is significantly different from the mesomorphic cluster (*p* < 0.05), **#** is significantly different from the tall meso-ectomorphic cluster (*p* < 0.05). **#** indicates a tendency for $\dot{\text{V}}$O_2__peak_ (*p* = 0.056). POTT, mean power during a 15-km time trial; $\dot{\text{V}}$O_2__*peak*_, peak oxygen uptake; PO_*peak*_, Wingate peak power; PO_*jump*_, squat-jump mean power.

Physical performance of the anthropometric clusters is presented in Figure 3. The mesomorphic cluster showed a higher sprint performance compared to the short and tall meso-ectomorphic clusters (PO_peak_: *p* = 0.023 and *p* = 0.022, respectively; PO_jump_: *p* = 0.001 and *p* < 0.001) and lower endurance performance (PO_TT_: *p* < 0.001 and *p* < 0.001; $\dot{\text{V}}$O_2__peak_: *p* < 0.001 and *p* < 0.001). Compared to the tall subgroup, the short meso-ectomorphic cluster demonstrated similar values for PO_peak_ (*p* = 0.987) and PO_TT_ (*p* = 0.211), but a higher PO_jump_ (*p* = 0.033) and tendency for a higher $\dot{\text{V}}$O_2__peak_ (*p* = 0.056). In sum, the mesomorphic cluster showed a higher sprint performance, whereas the meso-ectomorphic groups demonstrated a better endurance performance.

### Relationships Between Anthropometry and Physical Performance

Table 2 displays relationships between anthropometry and physical performance. High time-trial performance and $\dot{\text{V}}$O_2__peak_ were both associated with lean cyclists with small girths, a small frontal area, high ectomorphy, and low meso- and endomorphy. High PO_peak_ and PO_jump_ related to cyclists with larger skinfolds, larger girths, and a low frontal area and body surface area per body mass, whereas high jumping performance also related to less lean cyclists with a high meso- and endomorphy and low ectomorphy. Thus, anthropometric characteristics of body size, shape, and composition were significantly related to sprint and endurance performance in a group of sprint, pursuit, and road cyclists.

**TABLE 2:** Relationships between anthropometry and physical performance within a group of competitive sprint, pursuit, and road cyclists.

	**Correlation**				***p*-value**			
		
	**Endurance**		**Sprint**		**Endurance**		**Sprint**	
				
	**PO_TT_**	**$\dot{\text{V}}$O_2__peak_**	**PO_peak_**	**PO_jump_**	**PO_TT_**	**$\dot{\text{V}}$O_2__peak_**	**PO_peak_**	**PO_jump_**
**Basics**								
Height (m)	–0.05	–0.19	–0.08	–0.26	0.822	0.377	0.709	0.221
Body mass (kg)	–0.65^∗∗^	–0.75^∗∗^	0.30	0.35	0.001	<0.001	0.151	0.096
BMI (kg/m^2^)	–0.58^∗∗^	–0.60^∗∗^	0.34	0.51^∗^	0.003	0.002	0.103	0.012
**Somatotypes**								
Mesomorphy	–0.58^∗∗^	–0.53^∗∗^	0.31	0.53^∗∗^	0.003	0.008	0.146	0.008
Ectomorphy	0.56^∗∗^	0.49^∗^	–0.30	–0.65^∗∗^	0.005	0.016	0.154	0.001
Endomorphy	–0.58^∗∗^	–0.66^∗∗^	0.32	0.52^∗∗^	0.003	0.001	0.123	0.009
**Body composition**								
Lean body mass (%)	0.63^∗∗^	0.68^∗∗^	–0.05	−0.51^∗^	0.001	<0.001	0.806	0.013
Muscle mass (%)	0.63^∗∗^	0.72^∗∗^	–0.14	−0.48^∗^	0.001	<0.001	0.525	0.019
Body fat (%)	–0.63^∗∗^	–0.68^∗∗^	0.05	0.51^∗^	0.001	<0.001	0.806	0.013
**Body size**								
Body surface area	−0.49^∗^	–0.61^∗∗^	0.19	0.16	0.016	0.001	0.370	0.458
Frontal area	−0.49^∗^	–0.61^∗∗^	0.19	0.16	0.016	0.001	0.370	0.458
BSA/body mass ^∗^ 10^–3^	0.75^∗∗^	0.82^∗∗^	−0.41^∗^	–0.52^∗∗^	<0.001	<0.001	0.047	0.010
FA/body mass ^∗^ 10^–3^	0.75^∗∗^	0.82^∗∗^	−0.41^∗^	–0.52^∗∗^	<0.001	<0.001	0.047	0.010
**Lengths (cm)**								
Thigh	0.04	–0.10	–0.04	–0.30	0.856	0.644	0.863	0.161
Shank	–0.21	–0.30	–0.05	–0.20	0.314	0.160	0.811	0.349
**Skinfolds (mm)**								
Sum of six skinfolds	–0.59^∗∗^	–0.70^∗∗^	0.43^∗^	0.58^∗∗^	0.002	<0.001	0.038	0.003
Sum of eight skinfolds	–0.60^∗∗^	–0.71^∗∗^	0.46^∗^	0.59^∗∗^	0.002	<0.001	0.024	0.002
**Breadths (mm)**								
Humerus	−0.39^∗^	−0.47^∗^	0.31	0.19	0.060	0.020	0.134	0.382
Femur	–0.15	–0.27	0.22	–0.08	0.496	0.210	0.298	0.700
**Girths (cm)**								
Upper arm relaxed	–0.72^∗∗^	–0.82^∗∗^	0.40^∗^	0.55^∗∗^	<0.001	<0.001	0.050	0.005
Waist	–0.70^∗∗^	–0.68^∗∗^	0.47^∗^	0.46^∗^	<0.001	<0.001	0.020	0.025
Gluteal	–0.67^∗∗^	–0.76^∗∗^	0.38^∗^	0.41^∗^	<0.001	<0.001	0.067	0.048
Thigh	–0.65^∗∗^	–0.77^∗∗^	0.33	0.43^∗^	0.001	<0.001	0.115	0.036
Calf	–0.54^∗∗^	−0.47^∗^	–0.07	0.26	0.006	0.021	0.743	0.228

*All performance measures were expressed relative to the cyclist’s body mass. ^∗^ significant correlation, *p* < 0.05. ^∗∗^ significant correlation, *p* < 0.01. ^∗^ indicates tendencies, *p* < 0.10. BMI, body mass index; BSA, body surface area; FA, frontal area; PO_*TT*_, mean power during a 15-km time trial; $\dot{\text{V}}$O_2__*peak*_, peak oxygen uptake; PO_*peak*_, Wingate peak power; PO_*jump*_, squat-jump mean power.*

## Discussion

This study shows how *k*-means clustering divided sprint, pursuit, and road cyclists into three distinct anthropometric clusters with differing physical performance. The mesomorphic cluster included all sprinters and demonstrated a higher sprint performance, whereas the short and tall meso-ectomorphic clusters of pursuit and road cyclists presented higher endurance performance. Anthropometric measures were also significantly related to performance. A high endurance performance was associated with a lean ectomorph physique with small girths and a small frontal area, whereas a high sprint performance related to cyclists with larger skinfolds, larger girths, and a low frontal area per body mass.

### Anthropometry-Dependent Specialization

Currently, anthropometric characteristics are commonly reported for predefined groups of athletes of a specific sports specialization. However, it is unknown whether a machine-learning approach – grouping athletes based on individual anthropometry using multiple anthropometric dimensions and independent of sports specialization – will reveal clusters of athletes that have a similar anthropometry and compete in the same sports discipline. Using unsupervised machine learning, we uncovered three clusters based on the athletes’ anthropometric characteristics. The mesomorphic cluster included all sprinters with a favorable somatotype for strength and speed performance, similar to that of elite [endo-, meso-, and ectomorphy: 2.5, 5.2, and 2.4 (White et al., 1982; McLean and Parker, 1989)] and Olympic track sprinters [1.8, 5.2, and 2.4 (Garay et al., 1974)]. The body size profile of our sprinters was comparable to that of Olympic track sprinters (Craig and Norton, 2001). Nonetheless, our sprinters were not as lean as elite track sprinters, illustrated by their higher sum of skinfolds and endomorphy (Garay et al., 1974; Foley et al., 1989), which may hamper cycling performance due to increased energetic costs to acceleration, rolling friction, and aerodynamic resistance. Thus, all cyclists of the mesomorphic cluster competed in track sprint disciplines and had a similar body size and shape to that of elite track sprinters.

The short and tall meso-ectomorphic clusters included pursuit and road cyclists, with somatotypes that favored endurance performance. These results confirm the trend for a higher ectomorphy and lower mesomorphy in more endurance-type cyclists (Garay et al., 1974; Foley et al., 1989; McLean and Parker, 1989). Cyclists in both clusters had a relatively low body fat percentage (∼9%), comparable to that of professional road cyclists (Mujika and Padilla, 2001). This is beneficial for successful performance, as body fat adds to body mass but not to power-producing capabilities (Craig and Norton, 2001). The meso-ectomorphic clusters mainly differed in body size; cyclists in the short cluster were significantly smaller, shorter, and lighter. These cyclists were not necessarily very short (∼180 cm), but shorter than average Dutch males, which are the world’s tallest people (Stulp et al., 2015). Smaller cyclists minimize the influence of aerodynamic resistance, giving them a competitive edge on most terrains, specifically during uphill climbing (Padilla et al., 1999; Lucía et al., 2000). Larger cyclists, however, minimize the energy costs to aerodynamic friction per body mass, giving them an advantage on level roads (Mujika and Padilla, 2001). Interestingly, the body size of the short cluster was remarkably similar to that of all-terrain road cyclists and the tall cluster matched the body size of flat-terrain road cyclists (Padilla et al., 1999).

Anthropometric clustering showed that all sprinters were allocated to one cluster, whereas pursuit and road cyclists were not assigned to separate clusters. Our findings demonstrate that it is difficult to distinguish pursuit and road cyclists based on their individual anthropometry, which corresponds to previous literature reporting similar anthropometric characteristics for pursuit and road cyclists (Garay et al., 1974; Foley et al., 1989). Nonetheless, short and tall endurance-type clusters did match the anthropometry of two other cycling specializations, that of all-terrain and flat-terrain road cyclists. Therefore, our clustering results did (partially) confirm existence of anthropometry-dependent specialization.

### Physical Performance

To gain more insight in how physical performance differs between groups of athletes with similar individual anthropometry, we also assessed the sprint and endurance performance of each cluster. To our knowledge, actual differences in sprint and endurance performance between anthropometric clusters have not yet been assessed. According to current literature (Gabriel and Zierath, 2017), anthropometry of our mesomorphic cluster was beneficial for strength and speed performance, whereas anthropometry of the meso-ectomorphic clusters favored endurance performance. We now show that performance differences between anthropometric clusters are in line with their anthropometric pre-dispositions, confirming higher sprint performance in the mesomorphic cluster and higher endurance performance in both meso-ectomorphic clusters (Figure 3).

The two endurance-type clusters revealed small, but unforeseen performance differences. $\dot{\text{V}}$O_2__peak_ was ∼5 mL kg^–1^ higher in the short cluster (*p* = 0.056), whereas PO_TT_ was similar between both clusters. These findings were particularly consistent with performance differences between all-terrain and flat-terrain cyclists (Padilla et al., 1999) and may relate to body size differences. Previous literature showed that smaller cyclists had ∼12.5% higher $\dot{\text{V}}$O_2__peak_ and ∼11% higher body surface-to-mass ratios compared to larger cyclists, but similar $\dot{\text{V}}$O_2_-values at submaximal intensities (Swain et al., 1987). Also in our study, $\dot{\text{V}}$O_2__peak_ and BSA-to-mass ratios were proportional and strongly related (*r* = 0.82), possibly due to the influence of surface area-to-mass ratio on cardiovascular variables (Mitchell et al., 1992). Therefore, it is likely that the higher $\dot{\text{V}}$O_2__peak_ in the short cluster was explained by their higher BSA-to-mass ratio. For sprint performance, PO_peak_ was similar, but PO_jump_ was higher in the short cluster. The former result was expected, as percentage lean body mass was comparable between clusters and as similar relative peak power values (W/kg) have been reported for subjects with a different body mass but comparable proportion lean body mass (Maciejczyk et al., 2015). Conversely, in line with isometric downscaling (Bobbert, 2013), the short cluster was expected to produce less, not more, power per body mass during jumping push-off. Smaller animals produce lower power per body mass than larger animals, as they jump with higher accelerations due to their shorter body segments, which hampers build-up of active state and let muscles operate at unfavorably high velocities (Bobbert, 2013). Nonetheless, our smaller cyclists did not show this and may have compensated this disadvantage by their larger proportion of muscle mass. In brief, the small performance differences between meso-ectomorphic clusters were likely explained by differences in body size and/or composition.

Relationships between anthropometry and performance revealed that high endurance performance was associated with a lean ectomorph physique with small girths and a small frontal area. Lean body composition facilitates prolonged and efficient power production, as illustrated in triathletes (Knechtle et al., 2011). Ectomorph-shaped athletes with small girths are also assumed to have long and slender muscles. Such muscles are metabolically more efficient, as they avoid the negative effect of a large muscle physiological cross-sectional area on oxygen consumption during endurance performance (Van der Zwaard et al., 2018). The high sprint performance related to cyclists with larger skinfolds, larger girths, and a low frontal area per body mass. Mesomorph athletes with larger girths are assumed to have hypertrophied muscles. Such muscles generally have a large physiological cross-sectional area – induced by muscle-fiber hypertrophy – which contributes to high sprint performance (Van der Zwaard et al., 2018). The relationship with skinfolds was more surprising, but likely due to a suboptimal body composition of our sprinters. The higher body fat percentage may explain why peak power per body mass was lower in our cyclists with respect to elite track sprinters, even though their absolute sprint performance was the same (Dorel et al., 2005). Taken together, our results show that sprint and endurance performance correspond to the clusters’ anthropometric predispositions and highlight the value of interpreting physical performance in light of the athlete’s individual anthropometry.

### Data Science

Using unsupervised machine learning, we were able to distinguish three subgroups with a distinct anthropometry, which were formed independent of the athlete’s cycling specialization. Unsupervised machine-learning techniques use unlabeled data (i.e., data without defined categories or groups) to learn and identify common relationships within the data. Clustering algorithms use these commonalties to divide the data into meaningful subgroups based on similarities in their individual subject characteristics (e.g., anthropometry). On the other hand, supervised machine-learning techniques may also be used to classify athletes, but these require labeled data with pre-defined subgroups (e.g., sports specialization). Therefore, unsupervised clustering algorithms are preferred, as these divide athletes into subgroups solely based on anthropometry and independent of the athlete’s sports specialization.

While performing *k*-means clustering optimization, several assumptions and considerations should be taken into account. *K*-means clustering operates under the assumptions that clusters should be spherical (circular and clearly separated) and of similar size. Both assumptions were met in this study. As for considerations, firstly, features should be standardized to *Z*-scores during pre-processing, as no single feature is more important than another. Secondly, all anthropometric dimensions should have the same number of variables to guarantee an equal contribution of dimensions to the formation of subgroups (i.e., three features for body size, shape, and composition). Nonetheless, the same clusters were obtained when clustering without sum of skinfolds and BSA. Thirdly, clustering algorithms require researchers to specify the number of clusters in advance. Note that this could affect cluster validity, and therefore, careful determination of the optimal number of clusters using validity criterions is warranted (Charrad et al., 2014; Scrucca et al., 2016). Lastly, for cluster stability, it is recommended to repeat the clustering procedure several times with different randomly chosen initial cluster centers (e.g., 25 starting partitions per trial). While fulfilling these considerations, we tested cluster stability by repeating the *k*-means algorithm for 1000 subsequent trials. Results presented the same clusters in every trial (obtained within three iterations), confirming stable anthropometric clusters in the present study. When taking these considerations into account, novel machine-learning clustering algorithms enable grouping of athletes based on their individual anthropometry using an integrative approach of multiple anthropometric dimensions, which provides new perspectives on anthropometry-dependent sports specialization.

### Practical Applications

Data science provides scientists with new tools for data analytics in sports. Here, we show that unsupervised machine learning divides cyclists into three anthropometric clusters with distinct differences in body size, shape, and composition, and revealed that sprint and endurance performance of clusters matched their anthropometric predispositions. Clustering may help athletes and coaches to discover how athletes match their sports discipline with their individual anthropometry. Future studies may also perform anthropometric clustering with a larger sample of cyclists competing in all cycling specializations.

## Conclusion

In this study, we show that unsupervised machine learning enables clustering of athletes based on their individual anthropometry using an integrative approach of multiple anthropometric dimensions. *K*-means clustering revealed a mesomorphic cluster of sprinters with a high sprint performance and short and tall meso-ectomorphic clusters of pursuit and road cyclists with a high endurance performance. Our clustering results did confirm anthropometry-dependent specialization for sprint- and endurance-type cyclists, whereas clusters distinguished between short and tall endurance-type cyclists (that matched the anthropometry of all-terrain and flat-terrain road cyclists) rather than pursuit and road cyclists. Machine-learning algorithms therefore provide new insights in how athletes match their sports discipline with their individual anthropometry.

## Data Availability Statement

The datasets generated for this study are available on request to the corresponding author.

## Ethics Statement

The studies involving human participants were reviewed and approved by the Medical Ethics Committee of the VU Medical Center, Amsterdam, Netherlands (NL49060.029.14). The patients/participants provided their written informed consent to participate in this study.

## Author Contributions

SZ, CR, RJ, and JK conceived and designed the work, acquired, analyzed, and interpreted the data, and drafted and revised the manuscript.

## Conflict of Interest

The authors declare that the research was conducted in the absence of any commercial or financial relationships that could be construed as a potential conflict of interest.

## References

[B1] Bobbert M. F. (2013). Effects of isometric scaling on vertical jumping performance. *PLoS One* 8:e71209. 10.1371/journal.pone.0071209 23936494 PMC3731318

[B2] Brocherie F. Girard O. Forchino F. Al Haddad H. Dos Santos G. A. Millet G. P. (2014). Relationships between anthropometric measures and athletic performance, with special reference to repeated-sprint ability, in the Qatar national soccer team. *J. Sports Sci.* 32 1243–1254. 10.1080/02640414.2013.862840 24742185

[B3] Carter J. E. (1970). The somatotypes of athletes–a review. *Hum. Biol.* 42 535–569. 4928259

[B4] Chaouachi A. Brughelli M. Levin G. Boudhina N. B. B. Cronin J. Chamari K. (2009). Anthropometric, physiological and performance characteristics of elite team-handball players. *J. Sports Sci.* 27 151–157. 10.1080/02640410802448731 19051095

[B5] Charrad M. Ghazzali N. Boiteau V. Niknafs A. (2014). NbClust: an R package for determining the relevant number of clusters in a data set. *J. Stat. Softw.* 61 1–36. 10.18637/jss.v061.i06

[B6] Craig N. P. Norton K. I. (2001). Characteristics of track cycling. *Sports Med.* 31 457–468. 10.2165/00007256-200131070-00001 11428683

[B7] Dorel S. Hautier C. A. Rambaud O. Rouffet D. Van Praagh E. Lacour J.-R. (2005). Torque and power-velocity relationships in cycling: relevance to track sprint performance in world-class cyclists. *Int. J. Sports Med.* 26 739–746. 10.1055/s-2004-830493 16237619

[B8] Du Bois D. du Bois E. F. (1916). Clinical calorimetry: tenth paper a formula to estimate the approximate surface area if height and weight be known. *Arch. Intern. Med.* XVII, 863–871. 10.1001/archinte.1916.00080130010002. 2520314

[B9] Durnin J. V. Womersley J. (1974). Body fat assessed from total body density and its estimation from skinfold thickness: measurements on 481 men and women aged from 16 to 72 years. *Br. J. Nutr.* 32 77–97. 10.1079/BJN19740060 4843734

[B10] Foley J. P. Bird S. R. White J. A. (1989). Anthropometric comparison of cyclists from different events. *Br. J. Sports Med.* 23 30–33. 10.1136/bjsm.23.1.30 2730997 PMC1478660

[B11] Gabriel B. M. Zierath J. R. (2017). The limits of exercise physiology: from performance to health. *Cell Metab.* 25 1000–1011. 10.1016/j.cmet.2017.04.018 28467920

[B12] Garay A. L. de Levine L. Carter J. E. L. (1974). *Genetic and Anthropological Studies of Olympic Athletes.* Academic Press: Cambridge, MA.

[B13] Hartigan J. A. Wong M. A. (1979). Algorithm AS 136: A K-means clustering algorithm. *J. R. Stat. Soc. Ser. C Appl. Stat.* 28 100–108. 10.2307/2346830

[B14] Heath B. H. Carter J. E. L. (1967). A modified somatotype method. *Am. J. Phys. Anthropol.* 27 57–74. 10.1002/ajpa.1330270108 6049820

[B15] Knechtle B. Knechtle P. Rosemann T. (2011). Upper body skinfold thickness is related to race performance in male Ironman triathletes. *Int. J. Sports Med.* 32 20–27. 10.1055/s-0030-1268435 21110283

[B16] Lee R. C. Wang Z. Heo M. Ross R. Janssen I. Heymsfield S. B. (2000). Total-body skeletal muscle mass: development and cross-validation of anthropometric prediction models. *Am. J. Clin. Nutr.* 72 796–803. 10.1093/ajcn/72.3.796 10966902

[B17] Lucía A. Joyos H. Chicharro J. L. (2000). Physiological response to professional road cycling: climbers vs. time trialists. *Int. J. Sports Med.* 21 505–512. 10.1055/s-2000-7420 11071054

[B18] Maciejczyk M. Wiecek M. Szymura J. Szygula Z. Brown L. E. (2015). Influence of increased body mass and body composition on cycling anaerobic power. *J. Strength Cond. Res.* 29 58–65. 10.1519/JSC.0000000000000727 25353079

[B19] Marfell-Jones M. Olds T. Stewart A. Carter L. (2006). *International Standards for Anthropometric Assessment.* Potchefstroom: The International Society for the Advancement of Kinanthropometry.

[B20] McLean B. D. Parker A. W. (1989). An anthropometric analysis of elite Australian track cyclists. *J. Sports Sci.* 7 247–255. 10.1080/02640418908729845 2621762

[B21] Menaspà P. Rampinini E. Bosio A. Carlomagno D. Riggio M. Sassi A. (2012). Physiological and anthropometric characteristics of junior cyclists of different specialties and performance levels. *Scand. J. Med. Sci. Sports* 22 392–398. 10.1111/j.1600-0838.2010.01168.x 20807389

[B22] Mitchell J. H. Tate C. Raven P. Cobb F. Kraus W. Moreadith R. (1992). Acute response and chronic adaptation to exercise in women. *Med. Sci. Sports Exerc.* 24 S258–S265. 1625551

[B23] Mujika I. Padilla S. (2001). Physiological and performance characteristics of male professional road cyclists. *Sports Med.* 31 479–487. 10.2165/00007256-200131070-00003 11428685

[B24] Norton K. Olds T. (2001). Morphological evolution of athletes over the 20th century: causes and consequences. *Sports Med.* 31 763–783. 10.2165/00007256-200131110-00001 11583103

[B25] Norton K. Olds T. Scott O. Craig N. P. (1996). “Anthropometry and sports performance,” in *Anthropometrica: A Textbook of Body Measurement for Sports and Health Courses* eds Norton K. Olds T. (Sydney, NSW: University of New South Wales Press), 287–364.

[B26] Padilla S. Mujika I. Cuesta G. Goiriena J. J. (1999). Level ground and uphill cycling ability in professional road cycling. *Med. Sci. Sports Exerc.* 31 878–885. 10.1097/00005768-199906000-00017 10378916

[B27] Pison G. Struyf A. Rousseeuw P. J. (1999). Displaying a clustering with CLUSPLOT. *Comput. Stat. Data Anal.* 30 381–392. 10.1016/S0167-9473(98)00102-9

[B28] Scrucca L. Fop M. Murphy T. B. Raftery A. E. (2016). mclust 5: clustering, classification and density estimation using gaussian finite mixture models. *R J.* 8 289–317. 27818791 PMC5096736

[B29] Stulp G. Barrett L. Tropf F. Mills M. (2015). Does natural selection favour taller stature among the tallest people on earth? *Proc. R. Soc. B Biol. Sci.* 282:20150211. 10.1098/rspb.2015.0211 25854890 PMC4426629

[B30] Swain D. P. Coast J. R. Clifford P. S. Milliken M. C. Stray-Gundersen J. (1987). Influence of body size on oxygen consumption during bicycling. *J. Appl. Physiol.* 62 668–672. 10.1152/jappl.1987.62.2.668 3558226

[B31] Van der Zwaard S. de Ruiter C. J. Noordhof D. A. Sterrenburg R. Bloemers F. W. de Koning J. J. (2016). Maximal oxygen uptake is proportional to muscle fiber oxidative capacity, from chronic heart failure patients to professional cyclists. *J. Appl. Physiol.* 121 636–645. 10.1152/japplphysiol.00355.2016 27445298

[B32] Van der Zwaard S. van der Laarse W. J. Weide G. Bloemers F. W. Hofmijster M. J. Levels K. (2018). Critical determinants of combined sprint and endurance performance: an integrative analysis from muscle fiber to the human body. *FASEB J.* 32 2110–2123. 10.1096/fj.201700827R 29217665

[B33] White J. A. Quinn G. Al-Dawalibi M. Mulhall J. (1982). Seasonal changes in cyclists’ performance. Part II. the british olympic track squad. *Br. J. Sports Med.* 16 13–21. 10.1136/bjsm.16.1.13 7066610 PMC1858846

